# The complete chloroplast genome of hemi-parasitic *Pedicularis hallaisanensis* (Orobanchaceae)

**DOI:** 10.1080/23802359.2018.1437820

**Published:** 2018-02-12

**Authors:** Won-Bum Cho, Dong-Hyuk Lee, In-Su Choi, Jung-Hyun Lee

**Affiliations:** a Department of Biology Education, Chonnam National University, Gwangju, Republic of Korea;; b Baekdudaegan Biodiversity Conservation Division, Baekdudaegan National Arboretum, Bonghwa-gun, Republic of Korea;; c Department of Biological Sciences, Inha University, Incheon, Republic of Korea

**Keywords:** Chloroplast genome, hemi-parasite, *Pedicularis hallaisanensis*, phylogenetic analysis

## Abstract

We determined the complete chloroplast genome of *Pedicularis hallaisanensis* (Orobanchaceae), a hemi-parasitic perennial herb. This genome is 143,469 bp long and features a large single-copy region (81,664 bp) and a small single-copy region (12,203 bp), separated by two inverted-repeat regions (24,801 bp each). It contains 115 genes – 70 for coding, eight for rRNA, and 37 for tRNA. However, 11 *ndh* genes have been pseudogenized, truncated, or deleted. Our phylogenetic tree showed that these hemi-parasitic plants are sister to holo-parasitic genera within Orobanchaceae.

*Pedicularis hallaisanensis* Hurus. (Orobanchaceae) is a perennial herb that is endemic but endangered in Korea (Cho and Choi 2011). This species is root-hemi-parasitic, similar to other *Pedicularis* members. Although the chloroplast (cp) genomes of land plants are highly conserved (Jansen and Ruhlman 2012), the genomes of parasitic plants, such as those within Orobanchaceae, have experienced remarkable changes in size, structure, and gene contents (Xie et al. 2012; Wicke et al. 2013). Here, we report the complete cp genome of the hemi-parasitic *P. hallaisanensis* and examine its phylogenetic position within Orobanchaceae.

Plant material of *P. hallaisanensis* was collected from Mt. Halla on Jeju Island, Korea (N 33°21′49″, E 126°31′44″), and a voucher specimen was deposited in the herbarium of Inha University (Cho. 98454). Genomic DNA was extracted from young, silica gel-dried leaves through a protocol that used a DNeasy Plant Mini Kit (Qiagen, Seoul, Korea). The extracted DNA was sequenced via the Mi-Seq Illumina platform (LAS, Seoul, Korea), generating 11,213,054 raw reads. The cp genome was assembled using Geneious 10.2.3 software (Kearse et al. 2012) and reference sequences of *P*. *ishidoyana* (NC029700) and *Lathraea squamaria* (NC027838). Gene annotations were made with the DOGMA program (Wyman et al. 2004), and were manually corrected for start and stop codons and for intro/exon boundaries. Using default parameters and 1000 bootstrap replicates, we constructed a phylogenetic tree based on maximum likelihood (ML) analysis that included RAxML-HPC v.8 (Stamatakis 2014), which is available from the CIPRES gateway (Miller et al. 2010).

The complete cp genome of *P. hallaisanensis* (GenBank: MG770330) is 143,469 bp long, and shows a quadripartite structure with two inverted-repeat regions (24,801 bp each) that separate a large single-copy region (81,664 bp) and a small single-copy region (12,203 bp). This cp genome contains 115 genes, i.e. 70 for coding, eight for ribosomal RNA, and 37 for transfer RNA. All 11 of its *ndh* genes have been pseudogenized (*ndh*B, *ndh*E, *ndh*H, *ndh*G, *ndh*J, and *ndh*K), truncated (*ndh*A and *ndh*D), or deleted (*ndh*C, *ndh*F, and *ndh*I), a phenomenon also seen with other parasitic species (Krause 2011). Our phylogenetic tree includes 61 coding genes extracted from the cp genome of *P. hallaisanensis*, plus 18 related species downloaded from the NCBI database in Lamiales. The ML tree demonstrates that *Pedicularis* is sister to *Lathraea squamaria*, based on their high bootstrap values (Figure 1). These new phylogenetic data provide insight into the evolutionary progress of Orobanchaceae.

**Figure 1.**
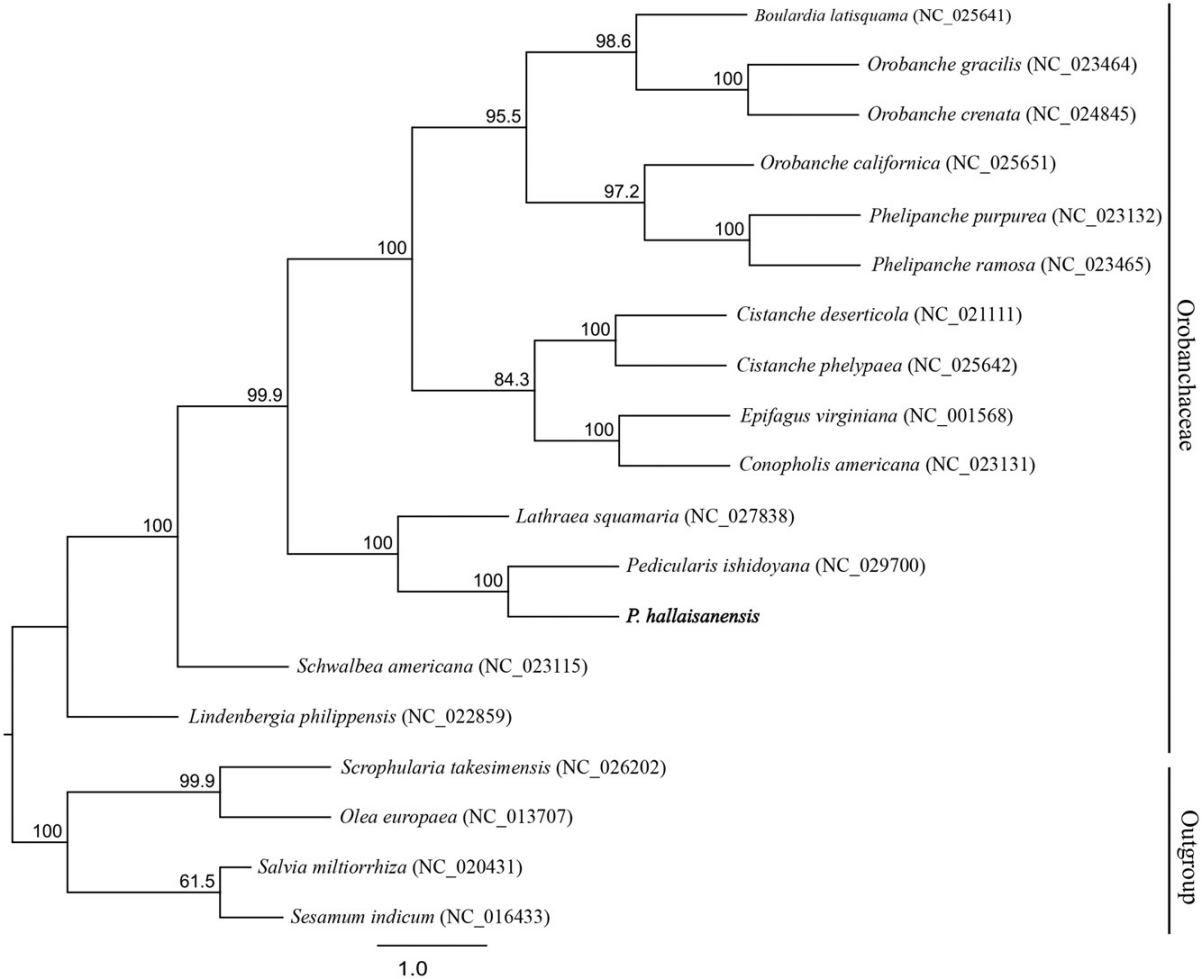
Phylogenetic tree constructed with RAxML, using dataset of 61 concatenated protein-coding regions from 19 chloroplast genomes. Numbers above nodes indicate bootstrap values (1000 replicates) > 50%.
